# *Bacillus velezensis* CE 100 Inhibits Root Rot Diseases (*Phytophthora* spp.) and Promotes Growth of Japanese Cypress (*Chamaecyparis obtusa* Endlicher) Seedlings

**DOI:** 10.3390/microorganisms9040821

**Published:** 2021-04-13

**Authors:** Jae-Hyun Moon, Sang-Jae Won, Chaw Ei Htwe Maung, Jae-Hyeok Choi, Su-In Choi, Henry B. Ajuna, Young Sang Ahn

**Affiliations:** 1 Department of Forest Resources, College of Agriculture and Life Sciences, Chonnam National University, Gwangju 61186, Korea; mjh132577@naver.com (J.-H.M.); lazyno@naver.com (S.-J.W.); cjh960728@gmail.com (J.-H.C.); suin917@naver.com (S.-I.C.); ajunahenry@mmu.ac.ug (H.B.A.); 2 Division of Agricultural and Biological Chemistry, Institute of Environmentally Friendly Agriculture, College of Agriculture and Life Sciences, Chonnam National University, Gwangju 61186, Korea; chaweihtwemaung@gmail.com

**Keywords:** forest seedling production, antagonistic bacteria, lytic enzymes, phytopathogenic oomycetes, auxin, plant development, biocontrol agent

## Abstract

Root rot diseases, caused by phytopathogenic oomycetes, *Phytophthora* spp. cause devastating losses involving forest seedlings, such as Japanese cypress (*Chamaecyparis obtusa* Endlicher) in Korea. Plant growth-promoting rhizobacteria (PGPR) are a promising strategy to control root rot diseases and promote growth in seedlings. In this study, the potential of *Bacillus velezensis* CE 100 in controlling *Phytophthora* root rot diseases and promoting the growth of *C. obtusa* seedlings was investigated. *B. velezensis* CE 100 produced β-1,3-glucanase and protease enzymes, which degrade the β-glucan and protein components of phytopathogenic oomycetes cell-wall, causing mycelial growth inhibition of *P. boehmeriae*, *P. cinnamomi*, *P. drechsleri* and *P. erythoroseptica* by 54.6%, 62.6%, 74.3%, and 73.7%, respectively. The inhibited phytopathogens showed abnormal growth characterized by swelling and deformation of hyphae. *B. velezensis* CE 100 increased the survival rate of *C. obtusa* seedlings 2.0-fold and 1.7-fold compared to control, and fertilizer treatment, respectively. Moreover, *B. velezensis* CE 100 produced indole-3-acetic acid (IAA) up to 183.7 mg/L, resulting in a significant increase in the growth of *C. obtusa* seedlings compared to control, or chemical fertilizer treatment, respectively. Therefore, this study demonstrates that *B. velezensis* CE 100 could simultaneously control *Phytophthora* root rot diseases and enhance growth of *C. obtusa* seedlings.

## 1. Introduction

The Japanese cypress (*Chamaecyparis obtusa* Endlicher) is a conifer of the cypress family (*Cupressaceae*), cultivated in many parts of Asia, including Japan, China and Korea [1,2,3,4,5]. The wood of *C. obtusa* has been used for centuries for furniture and the construction of temples and other traditional buildings due to its natural fragrance, good quality and high durability in outdoor conditions [1,4,5]. In particular, *C. obtusa* has been commercially used in the production of perfumes, cosmetics, soap, toothpaste, and disinfectants in Korea, due to its fresh fragrance [4,5]. In Korea, the afforestation areas of *C. obtusa* has been gradually increased and it ranked the first, accounting for 28.9% of total seedlings produced in 2020 [6]. The intensive production of tree seedlings often requires the use of chemical fertilizers to improve nutritional balance for successful seedling growth [7]. However, the conventional system of forest seedling production often uses excessive chemical fertilizers. This leads to plant defects, increases disease susceptibility, accelerates soil erosion and reduces efficiency of nutrient re-uptake and soil fertility [8,9,10]. Moreover, intensive nitrogen fertilization has potential for increasing the proportion of phytopathogens in the soil, which could negatively affect tree seedlings [9].

**Figure 1:**
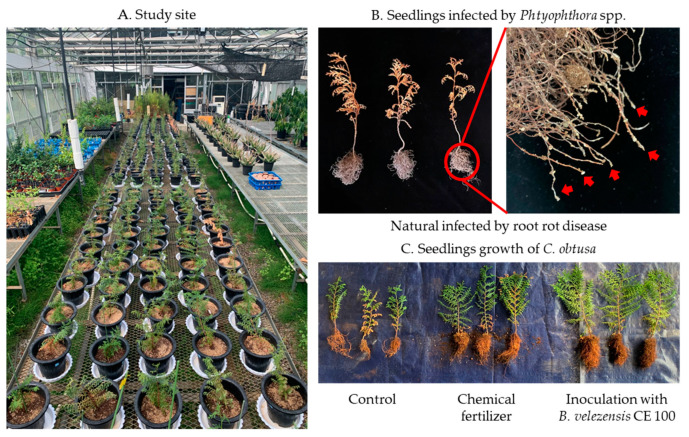
Study area in an experimental greenhouse at the forest nursery (**A**); seedlings infected by *Phytophthora* spp. (**B**); seedlings growth of control conditions, chemical fertilizer treatment and bacterial inoculation with *B. velezensis* CE 100 (**C**).

In recent years, *Chamaecyparis*, a member of *C. obtuse*, which is susceptible to oomycete pathogens, has faced an increasing challenge of *phytophthora* root rot diseases during forest seedling production [8,11,12]. The genus *Phytophthora* has over 150 species and commonly occurs in water-saturated soils [13,14]. It is the most devastating causal agent of root and stem rot, causing discoloration, necrotic leaves and wilting of new growths, leading to death of forest seedlings [8,11,13,14,15]. *Phytophthora* root rot diseases in *C. obtusa* seedlings cause yellowing and brown colorations from the lower leaves, which extends to the upper canopy, leading to complete wilt. Similar symptoms were observed in this study (Figure 1B). The deciduous oblong shaped sporangia, produced by *Phytophthora* spp., induce root rot diseases by releasing large numbers of zoospores that are attached to the tips of young roots where they encyst, germinate and infect other roots [11,16,17,18]. After infection, the oomycete quickly colonizes the root system, causing death of plant tissue due to necrosis in the inner bark [12,18]. Ultimately, the entire canopy of infected seedlings progressively develops foliage symptoms, such as pale green, yellow and light brown coloration, leading to the death of the entire tree [11,12,16]. Due to the highly destructive nature of these pathogens, an effective disease management strategy is essential for ensuring healthy forest seedlings.

The use of chemical fungicides is the most widely used control method for suppressing the invasion of *Phytophthora* spp. in forest seedlings. However, frequent chemical fungicides cause environmental contamination and could lead to the development of resistant strains [19,20,21]. In addition, such chemicals also kill useful soil organisms and beneficial microbes in the root zone. Excessive chemical use also reduces soil fertility, pollutes water resources and consequently cause harmful effects on human health [21,22]. Due to the increasing concern about the environment and human health, biological control of plant diseases has received increased focus [19,21]. Among the various methods, the use of plant growth-promoting rhizobacteria (PGPR) has attracted attention of many researchers. PGPR can control pathogenic oomycetes and increase plant growth [23,24,25,26,27,28]. Moreover, PGPR are well-known non-toxic bacteria that enhance soil fertility without causing negative effects on human health and the environment [28]. In addition, PGPR can effectively boost plant health by limiting the growth of plant pathogens through the production of antagonistic substances, antibiotics and cell wall degrading enzymes [25,26,27,28]. Lytic enzymes, such as β-1,3-glucanase and protease produced by PGPR are key players in the degradation of oomycete cell walls, which are mainly composed of β-glucan and protein [23,25,26,28,29]. On the other hand, PGPR provides plants with phytohormones, such as auxins, gibberellins and cytokinins, which regulate plant growth [24,28,30,31,32,33,34,35,36]. In particular, PGPR secretes auxins, such as indole-3-acetic acid (IAA), which improve the growth of shoots and roots by increasing root surface area, which in turn, promote nutrient uptake [23,24,28,30,31,32,33,34,35,36,37,38,39]. Specifically, *Bacillus* species possess significant inhibitory activity against various phytopathogens, including *Phytophthora capsici* [40,41] and *Phytophthora drechsleri* [42]. In addition, *Bacillus* species are known to produce auxins, which promote plant growth [31,32,33,34,35]. 

In the field of forestry, the production of high-quality forest seedlings that are infection-free is the most important factor for a successful nursery [43,44]. Well-developed, healthy seedlings perform better than small, weak seedlings after transplanting, especially under competitive conditions with forest weeds [45]. However, increasing the production of high-quality seedlings, while reducing the use of synthetic chemical fertilizers and fungicides, is a major challenge. Many PGPR species have been reported to improve plant growth and to control root rot pathogens. However, the potential of PGPR in the management of *Phytophthora* root rot diseases and growth promotion of *C. obtusa* seedlings has not been described. For sustainable production of high-quality cypress seedlings, it is important to understand the inhibitory mechanisms employed by PGPR against phytopathogens, as well as the biochemical interactions that influence seedling development. Therefore, the objective of this study was to investigate the effects of *Bacillus velezensis* CE 100 on the inhibition of *Phytophthora* spp., and the subsequent management of *Phytophthora* root rot diseases as well as growth promotion of *C. obtusa* seedlings.

## 2. Materials and Methods

### 2.1. Preparation of B. velezensis CE 100 and Phytophthora spp.

Bacterial strains *B. velezensis* CE 100, used in this study, were isolated from pot soils of tomato plants [46]. Then, *B. velezensis* CE 100 was streaked onto tryptone soy agar (TSA) medium and inoculated at 30 °C for 3 days. To examine the growth conditions and activities of lytic enzymes (β-1,3-glucanase and protease) produced by strain *B. velezensis* CE 100, pink broth (PB) (pink fertilizer (NPK 20-20-20) 3 g, KH_2_PO_4_ 0.2 g, MgSO_4_ 0.2 g, NaCl 0.1 g, sucrose 3 g, chitin powder 0.5 g and yeast extract 0.6 g in 1 L distilled water) medium was pre-inoculated with a loopful of *B. velezensis* CE 100 colonies and cultured at 30 °C in a shaking incubator (H1012 Incu-Shaker, Benchmark Scientific, Inc., Edison, NJ, USA) at 120 rpm for 3 days [30,31,32,47]. Then, 200 µL of this pre-inoculated bacterial culture broth (10^5^ colony-forming unit (CFU)/mL) was inoculated into 200 mL of PB broth, followed by incubation at 30 °C, with shaking at 120 rpm in a shaking incubator for 10 days. The experiment was replicated three times. During incubation, samples were collected every day for 10 days. After serial dilution, samples were spread on TSA plates and incubated at 30 °C for 1 day. The number of cells were counted as CFUs for each incubation day to determine the growth pattern of strain *B. velezensis* CE 100. 

### 2.2. Quantitative Analysis of Lytic Enzymes

Protease activity was determined following the method described by Ghorbel-Frikha et al. [48]. Briefly, Tris buffer (100 mM) containing 2 mM CaCl_2_ and 1% casein was prepared and adjusted to pH 8.0. A reaction mixture, containing 50 µL of bacterial supernatant and 950 µL of Tris buffer, was incubated at 60 °C for 15 min. Then 500 µL of 20% trichloroacetic acid was added to terminate the reaction. The mixture was centrifuged at 13,000 rpm for 15 min. The absorbance of the supernatant containing acid-soluble proteins was measured at 280 nm using a UV spectrophotometer (UV-1650PC, Shimadzu, Kyoto, Japan). One unit of protease activity was defined as the amount of enzyme that liberated 1 µg of tyrosine per minute.

The activity of β-1,3-glucanase was determined using the method described by Liang et al. [49]. Briefly, a reaction mixture containing 50 μL of bacterial supernatant, 50 μL of laminarin (10 mg/mL) and 400 μL of 50 mM sodium acetate buffer (pH 5.0) was incubated at 37 °C for 1 h. The reaction was stopped by adding 1.5 mL of 3,5-dinitrosalicylic acid (DNS) reagent and boiled in a water bath for 5 min. Absorbance at 550 nm was then measured to determine the concentration of reducing sugars. One unit of β-1,3-glucanase activity was defined as the amount of enzyme that catalyzed the release of 1 μmol of glucose per hour at 37 °C.

### 2.3. Anti-Oomycete Activity of B. velezensis CE 100 Against Phytophthora spp.

During seedling growth of *C. obtusa*, root rot diseases symptoms, such as drying of leaves and eventual death of the seedlings, were observed (Figure 1B). Phytopathogenic oomycete isolates were isolated from diseased roots of 5 seedlings in each treatment. The isolates were identified as *P. boehmeriae*, *P. cinnamomi*, *P. drechsleri*, *P. erythoroseptica* based on 18s RNA gene sequence. Consequently, the oomycete pathogens *P. boehmeriae* (KACC 44718), *P. cinnamomi* (KACC 40182), *P. drechsleri* (KACC 40198) and *P. erythoroseptica* (KACC 40712) with known virulence against *C. obtusa* were obtained from Korean Agriculture Culture Collection (KACC; Suwon, Korea) for in vitro antagonistic assay with *B. velezensis* CE 100. These four phytopathogenic oomycetes were cultured in potato dextrose agar medium at 25 °C for 10 days. Antagonistic activities *of B. velezensis* CE 100 against these four phytopathogenic oomycetes were determined using the dual culture method. The bacterial antagonist was streaked onto one side of each agar plate. Then, a 5 mm plug of each phytopathogenic oomycete was made using a sterile cork borer and placed on the other side of the inoculated plate. Plates were then incubated at 25 °C. Depending on the growth rate of each pathogen, days of incubation were different: *P. cinnamomi*, 7 days; *P. boehmeriae*, *P. drechsleri* and *P. erythoroseptica*, 10 days. A plate inoculated with each oomycete pathogen alone was used as the control. The experiment was repeated three times with three replications per treatment. The growth inhibition of oomycete pathogens was calculated using the following formula: Inhibition (%) = [(α − β)/α] × 100, where α was the radial growth of phytopathogenic oomycete on the control plate and β was the radial growth of phytopathogenic oomycete on the dual culture plate [24,26,27,28].

To examine the effects of *B. velezensis* CE 100 on hyphal morphologies of oomycete pathogens, a small piece of phytopathogenic oomycete hyphae at the boundary of the pathogen colony co-inhabited with *B. velezensis* CE 100 was taken and observed for hyphal deformation and degradation. Phytopathogenic oomycete mycelia were observed under a light microscope at 200× magnification (BX41TF Microscope, Olympus, Tokyo, Japan). All experiments for morphological observation of mycelia were performed in triplicate.

### 2.4. Indole-3-Acetic Acid (IAA) Production by B. velezensis CE 100

Quantitative analyses of IAA produced by *B. velezensis* CE 100 were performed using a UV spectrometric method, as described previously [50]. Briefly, *B. velezensis* CE 100 was cultured in a medium containing fertilizer 3 g, KH_2_PO_4_ 0.2 g, MgSO_4_ 0.2 g, NaCl 0.1 g, sucrose 3 g, chitin powder 0.5 g, yeast extract 0.6 g and 0.1 g L-tryptophan in 1 L distilled water. The culture was incubated at 30 °C in a shaking incubator at 140 rpm. Samples were taken every day from the day of inoculation. The samples were centrifuged at 12,000 rpm for 10 min at 4 °C. Then, 1 mL of the resulting supernatant was mixed with 2 mL of Salkowski’s reagent. Subsequently, the reaction mixture was incubated at room temperature in the dark for 25 min. The concentration of IAA in each sample was measured at 530 nm using a UV spectrometer (UV-1650PC, Shimadzu, Kyoto, Japan).

### 2.5. Experimental Conditions

The experiment was carried out with three replications in a greenhouse at Chonnam National University, Korea (approximately 35°17′ N latitude, 126°90′ E longitude) (Figure 1A). During the experiment, the temperature was maintained at 20–25 °C all day using a heating and cooling system and light was provided using natural light. The experiment was conducted using 2 years old *C. obtusa* seedlings purchased from a seedling company in October 2019. Each seedling was approximately 15 cm high, and the root collar diameter was 1.5 cm. The pots used were 20 cm in diameter and 25 cm in height. The pots were washed with 2% H_2_O_2_ for sterilization and dried before use [51]. Seedlings were planted in approximately 500 g of potted soil mixture (vermiculite, sand, red soil, and topsoil = 1:2:1:1) at the study site in October 2019. The following three treatment groups were used in this experiment: control (without fertilizer or bacteria), chemical fertilizer and *B. velezensis* CE 100 inoculation. For each treatment, 20 replicates were used, and each replicate was repeated 3 times with a 100 cm buffer zone between the blocks. A total of 180 seedlings were used in the experiment, with 60 seedlings for each treatment group. To avoid position effect, seedlings of each treatment were moved every month. At one month after planting, bacterial inoculum cultured at 30 °C for 7 days was applied to the root zone at a rate of 100 mL/seedling every 10 days. For chemical fertilizer, 3 g of pink fertilizer (NPK 20-20-20) in 1 L distilled water were applied to the root zone a rate of 100 mL/seedling every 10 days. Control seedlings received 100 mL of water per seedling every 10 days, without any bacteria or fertilizer. All treatments were applied from November 2019 to June 2020.

### 2.6. Analysis of Survival Rate of C. obtusa Seedlings

Survival rates of seedlings were surveyed from November 2019 to June 2020. Seedlings of *C. obtusa* were considered dead when their leaves and shoots were dried or absent. The survival rate was calculated as a percentage of surviving seedlings among total seedlings.

### 2.7. Determination of Seedling Growth Parameters

To determine seedling growth (root collar diameter and lengths of shoot and root) and seedling biomass (shoot and root dry weights), all live seedlings were taken to the Department of forest resources at Chonnam National University in June 2020. After carefully washing the root area to eliminate all media and debris, the root collar diameter and lengths of the shoots, and roots were measured, respectively. The shoots and roots were separated at the boundaries of the uppermost parts of root. To determine the biomass, the shoots and roots of each treatment were dried for 24 h in pre-weighed, moisture free paper bag at 105 °C, in a convection drying oven (VS-1202D4, Vision Scientific, Daejeon, Korea) and the dry weight measured [31].

### 2.8. Statistical Analysis

Statistical analyses were performed using SPSS (Statistical Package for the Social Sciences), version 25 (Armonk, NY, USA). All data were subjected to an analysis of variance (ANOVA). The mean values were compared using Fisher’s Least Significant Difference (LSD) test at *p* < 0.01.

## 3. Results

### 3.1. Inhibition of Root Rot Diseases Caused by Phytophthora spp. Using B. velezensis CE 100

#### 3.1.1. Growth Pattern of *B. velezensis* CE 100

**Figure 2:**
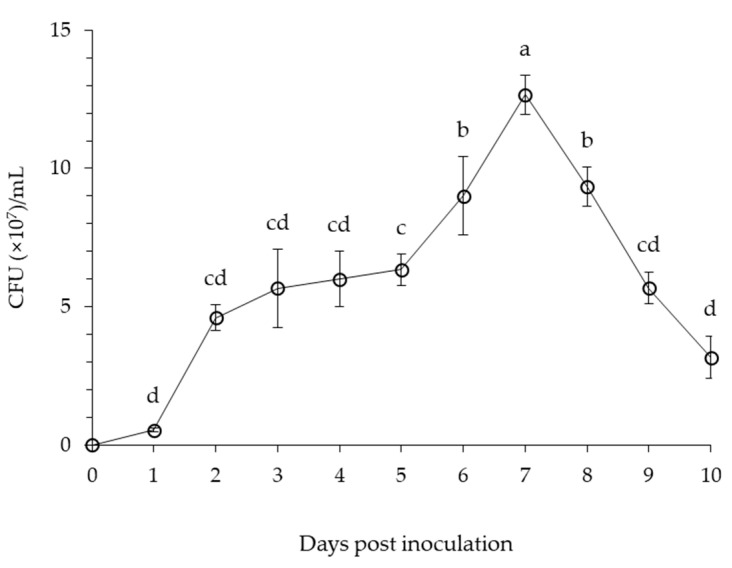
Cell growth curve of *B. velezensis* CE 100 in PB medium at 30 °C for 10 days. Error bars represent the standard deviation of three replications. Means with the same letter are not significantly different at *p* < 0.01 when compared using LSD test.

The growth of *B. velezensis* CE 100 increased slowly until 5 days after inoculation (Figure 2). Thereafter, the growth of *B. velezensis* CE 100 rapidly increased to a maximum value of 12.7 × 10^7^ CFU/mL at 7 days after inoculation Then, the growth rate of *B. velezensis* CE 100 steadily decreased from 8 days after inoculation until the end of the experimental period.

#### 3.1.2. Production of Lytic Enzymes by *B. velezensis* CE 100

**Figure 3:**
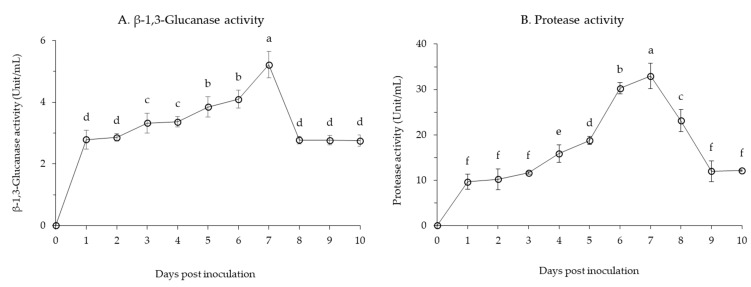
Changes in β-1,3-glucanase (**A**) and protease (**B**) activities in the culture supernatant of *B. velezensis* CE 100. Error bars represent the standard deviation of three replications. Means with the same letter are not significantly different at *p* < 0.01 when compared using LSD test.

The production of lytic enzymes, such as β-1,3-glucanase and protease activities, was examined from the bacterial culture of *B. velezensis* CE 100 (Figure 3). The β-1,3-glucanase activity increased steadily for 6 days, eventually reaching a maximum value of 5.2 unit/mL at 7 days (Figure 3A). The activity of β-1,3-glucanase stabilized after 8 days of incubation at about 3 unit/mL throughout the end of the study. In addition, *B. velezensis* CE 100 showed high protease activity during incubation. Protease activity rapidly increased to 10 unit/mL within 24 h after inoculation, and then increased gradually to 20 unit/mL in 5 days. Similar to the observed pattern in CFU, the activity of protease showed an exponential increase from 5 days to 7 days post inoculation, reaching a maximum value of 32.9 unit/mL (Figure 3B). The activity then decreased until to about 10 unit/mL by the end of the incubation period.

#### 3.1.3. Inhibition of Oomycete Pathogens by *B. velezensis* CE 100

**Figure 4:**
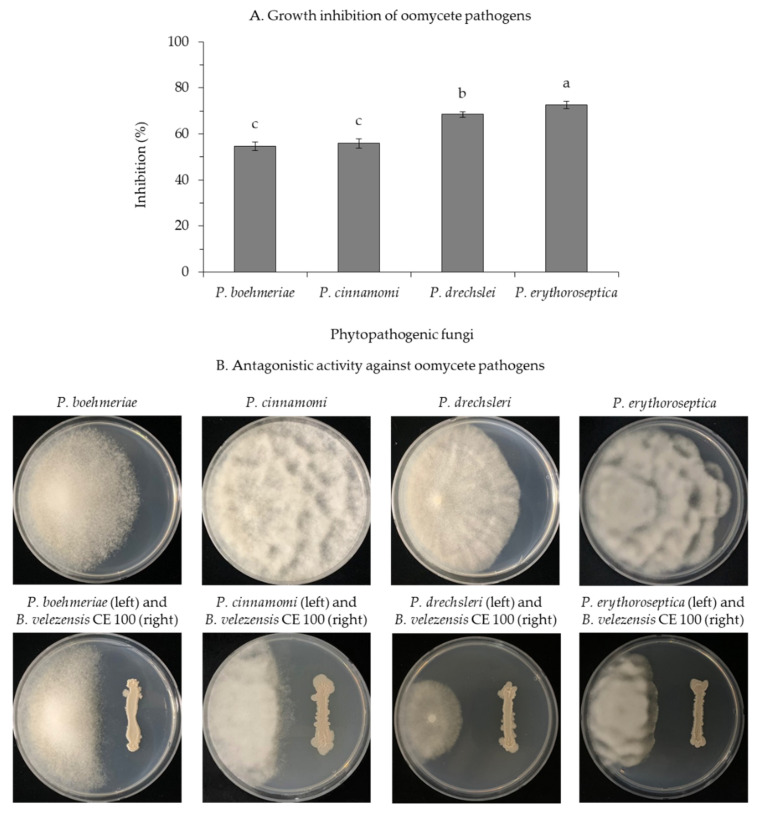
Inhibition effect of *B. velezensis* CE 100 on mycelial growth of *P. boehmeriae*, *P. cinnamomi*, *P. drechsleri* and *P. erythoroseptica* (**A**). Antagonistic activities of *B. velezensis* CE 100 against *P. boehmeriae*, *P. cinnamomi*, *P. drechsleri* and *P. erythoroseptica* (**B**) based on a dual culture method. Error bars represent the standard deviation of three replications. Means with the same letter are not significantly different at *p* < 0.01 when compared using LSD test.

**Figure 5:**
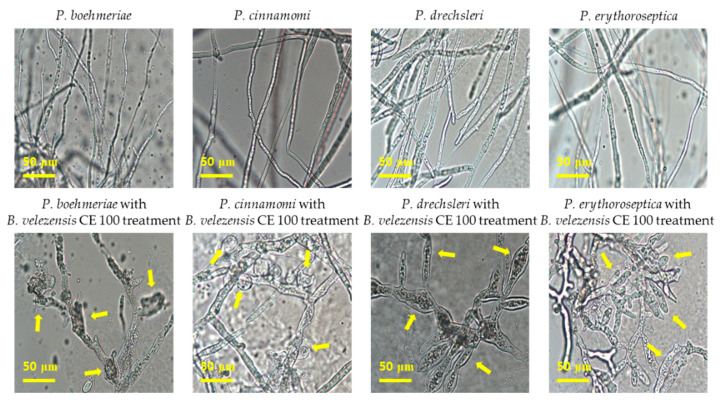
Inhibition effect of *B. velezensis* CE 100 on phytopathogenic hyphae morphologies of *P. boehmeriae, P. cinnamomi, P. drechsleri* and *P. erythoroseptica,* observed under a light microscope. On the top, the normal growth in the control group and at the bottom, the corresponding effect of *B. velezensis* CE 100. Arrows indicate hyphal alterations with swelling and deformation structures caused by *B. velezensis* CE 100.

Treatment with *B. velezensis* CE 100 showed strong anti-oomycete activity against phytopathogenic *Phytophthora species* used in this study (Figure 4). In a dual culture experiment, *B. velezensis* CE 100 inhibited the growth of each phytopathogenic oomycete as follows: *P. boehmeriae*, 54.6%; *P. cinnamomi*, 62.6%; *P. drechsleri*, 74.3%; and *P. erythoroseptica*, 73.7% (Figure 4A). Light microscopic observations of the hyphae of these phytopathogenic oomycete indicated normal hyphal growth in the control group and abnormal growths, such as swelling and deformation of the mycelia in *B. velezensis* CE 100 treatment group (Figure 5). 

#### 3.1.4. Survival Rate of *C. obtusa* Seedlings

The average survival rate of *C. obtusa* seedlings inoculated with *B. velezensis* CE 100 was higher than that of seedlings in the control group and chemical fertilizer treatment (Table 1). In particular, the average survival rate of *C. obtusa* seedlings inoculated with *B. velezensis* CE 100 (81.7%) in June 2020 was 2.0-fold or 1.7-fold higher than the survival rate of the seedlings in the control group (41.7%) and chemical fertilizer treatment (48.3%), respectively. There was no statistically significant difference between the survival rates in the control group and chemical fertilizer treatment.

### 3.2. Effect of B. velezensis CE 100 on Growth Promotion of C. obtusa Seedlings

#### 3.2.1. Indole-3-Acetic Acid (IAA) Production of *B. velezensis* CE 100

**Figure 6:**
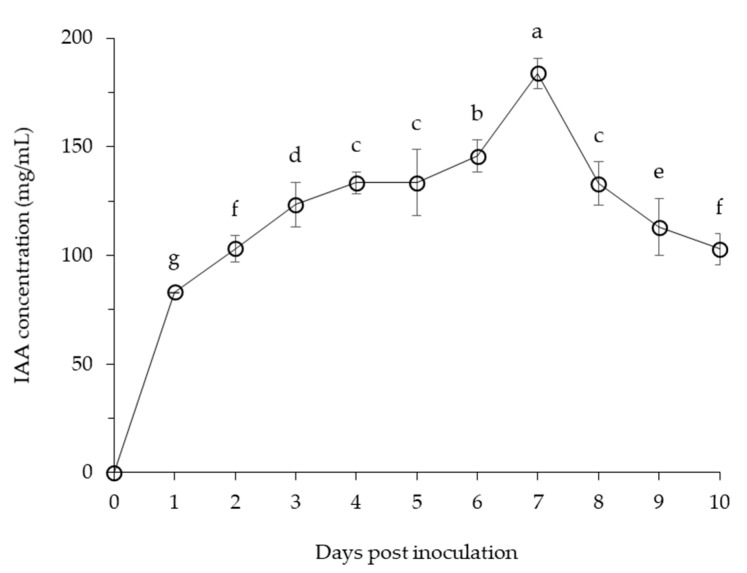
Changes in indole-3-acetic acid (IAA) concentration with *B. velezensis* CE 100. Error bars represent the standard deviation of three replications. The means with the same letter are not significantly different at *p* < 0.01 when compared using LSD test.

Incubation of *B. velezensis* CE 100 produced auxin, IAA during growth (Figure 6). The IAA concentration steadily increased for 6 days, eventually reaching a maximum value of 183.7 mg/L on 7 days. Thereafter, the IAA concentration decreased gradually to approximately 100 mg/mL at the end of the study.

#### 3.2.2. Growth and Biomass Yield of *C. obtusa* Seedlings

Significant increases in the growth (root collar diameter and lengths of shoot and root) and biomass (dry weights of shoot and root) were observed in *C. obtusa* seedlings, inoculated with *B. velezensis* CE 100, compared to both the control group and chemical fertilizer treatment (Table 2). The average root collar diameter, shoot length and root length of seedlings inoculated with *B. velezensis* CE 100 were 3.2 mm, 32.6 cm and 15.5 cm, respectively. Therefore, *B. velezensis* CE 100 increased collar diameter, shoot length and root length by 1.3, 1.4 and 1.5-folds, compared to the control group. The chemical fertilizer treatment also showed significantly higher root length compared to the control group, but no difference was observed with respect to the root collar diameter and shoot length.

In addition, the seedlings treated with bacterial inoculation had significantly higher biomass compared to the control group and chemical fertilizer. In particular, the dry weights of shoot and root of *C. obtusa* seedlings inoculated with *B. velezensis* CE 100 were 1.4, and 1.9-fold heavier than the control, respectively. The root dry weight was significantly higher in the chemical fertilizer treatment than in the control group, but no difference was observed in shoot dry weight. 

## 4. Discussion

### 4.1. Anti-Oomycete Activity of B. velezensis CE 100 against Phytophthora spp. and Survival Rate of Seedlings

Root rot diseases, caused by *Phytophthora* spp., are highly prevalent in forest soils and pose a major challenge in forest seedling production [12]. In this study, *Phytophthora* root rot diseases symptoms were observed in *C. obtusa* seedlings across the treatments, limiting the survival of seedlings (Table 1). Nonetheless, the survival rate of *C. obtusa* seedlings treated with *B. velezensis* CE 100 was 2.0 and 1.7-fold higher than that of the control group, and chemical fertilizer treatment, respectively (Table 1). Chemical fertilizer treatment and the control group were not statistically different in the survival rate of *C. obtusa* seedlings (Table 1). The use of chemical fertilizers increases the biomass of phytopathogens in the soil, which in turn increases the rate of seedling infection [52]. In the present study, *B. velezensis* CE 100 secreted cell wall-degrading enzymes such as β-1,3-glucanase and protease throughout the incubation period (Figure 3). Consequently, *B. velezensis* CE 100 showed strong inhibitory effects against the major oomycete pathogens associated to root rot diseases in *C. obtusa* seedlings: *P. boehmeriae*, 54.6%; *P. cinnamomi*, 62.6%; *P. drechsleri*, 74.3%; and *P. erythoroseptica*, 73.7%, in vitro (Figure 4). The hyphae of the phytopathogenic oomycetes in the group, treated with *B. velezensis* CE 100, showed an abnormal morphology, such as swelling and deformation under a light microscope (Figure 5). The cell wall of oomycete is composed of 80–90% glucan and 5–10% protein, which play an important role in maintaining cell wall integrity [53,54]. Cell wall degrading enzymes, produced by antagonistic bacteria, can exert a direct inhibitory effect on cell wall of many oomycete pathogens [25,26,28,29]. In particular, β-1,3-glucanase and protease are well-known for their ability to degrade and lyse the cell walls of oomycete [25,26,28,29]. The enzyme β-1,3-glucanase hydrolyzes substrates by sequentially cleaving glucose residues from the non-reducing end and cleaves linkages at random sites along the polysaccharide chain, releasing smaller oligosaccharides [55,56]. Protease activity involves the hydrolysis of one or more peptide bonds by adding water to liberate peptides or amino acids [57]. Consequently, these enzymes can degrade glycosidic bonds in the polysaccharide of cell wall, thereby reducing the growth of the cell wall, the tip of hyphae and the germination tube. They also cause morphological distortions, such as hyphal breakage and formation of sporadic, anomalous swelling along the surface of hyphae [25,26,54]. In a previous study, El-Sayed et al. [25] demonstrated that lytic enzymes produced by *Pseudomonas* species EA6 can hydrolyze of *Phytophthora parasitica* cell wall, which is consistent with results of this study. 

### 4.2. Effect of B. velezensis CE 100 on Growth Promotion of C. obtusa Seedlings

Besides biocontrol activity, PGPR provides plants with phytohormones, such as auxin that plays an important role in regulating plant growth and development by causing cell elongation through cell differentiation and expansion [23,24,34]. In the chemical fertilizer treatment, the root length and root dry weight of *C. obtusa* were significantly higher than that of the control group (Table 2). This is due to the fact, that seedlings develop roots to absorb nutrients and water before the shoots, and the chemical fertilizer treatment group developed the roots more through the absorption of excellent nutrients [58]. However, the root collar diameter, shoot length and shoot dry weight were not significantly different between the chemical fertilizer treatment and the control group (Table 2). This could be due to *phytophthora* root rot diseases, which cause necrosis in the inner bark of the root [12,18]. Necrosis reduces the ability of the root system to absorb and transport water and nutrients, which leads to retardation and consequently, the seedlings fail to attain the expected stage in shoot growth [59]. However, *C. obtusa* seedlings inoculated with *B. velezensis* CE 100 showed significant increases in growth and biomass, compared with the control group and chemical fertilizer treatment (Table 2). Therefore, *B. velezensis* CE 100 controlled *phytophthora* root rot in *C. obtusa* seedlings by secreting lytic enzymes such as β-1,3-glucanase and protease. This resulted in healthy seedlings with a well-developed root system to absorb and transport water and nutrients, which enhanced seedling growth. In addition, *B. velezensis* CE 100 secreted IAA into the medium to a concentration 183.7 mg/mL (Figure 6). The secretion of auxin could have enhanced shoot and root lengths (Table 2), as well as initiating lateral and adventitious root formation as observed (Figure 1C). Auxin can activate plasma membrane H^+^-ATPase proton pumps, which pump protons (H^+^) into the wall matrix, leading to wall acidification within pH between 4.5 and 6 [37,38,39]. The acidification of cell walls accelerates structural proteins such as EXPANSINs (EXPs), EXTENSINs (EXTs) and ARABINOGALACTAN PROTEINs (AGPs) [37,38,39]. As non-enzymatic wall-loosening proteins, EXPs can loosen the cell wall structure by breaking the hydrogen bond between cellulose microfibrils and hemicelluloses that connect them [37,38,39]. The activation of plasma membrane H^+^-ATPase causes hyperpolarization of the membrane potential and activation of voltage-dependent K^+^ inward, which transport potassium ions (K^+^) to the cytosol, thus, stimulating water (H_2_O) uptake and maintaining tensile stress [39]. As a result, the cell wall increases, causing cells to expand and become larger, which increases length of roots [39]. In addition, auxin provides a key signal during lateral root development, triggering the initial mitotic division of lateral root founder cells in the pericycle tissue to form lateral and adventitious roots [60,61]. Finally, root development can increase the surface area of root systems in contact with soil and lead to an increased ability for nutrient and water uptake, ultimately improving plant growth and yield. Park et al. [35] demonstrated that auxin produced by *Bacillus licheniformis* MH48 can increase dry weights of leaves and roots of *Camellia japonica* seedlings by 2.6, and 2.2-fold, respectively, compared to the control. Indeed, the results of this study clearly indicate that treatment with *B. velezensis* CE 100 bacterial inoculation significantly increased seedling growth and biomass production compared to chemical fertilizer treatment and the control group. The bacterial inoculation was particularly beneficial for improved root length and root collar diameter, which ultimately increased the surface area for nutrient uptake, leading to increased growth and biomass production in *C. obtusa* seedlings.

## 5. Conclusions

The results of the study suggest that, the antagonistic bacteria, *B. velezensis* CE 100 produces lytic enzymes, such as β-1,3-glucanase and protease. These lytic enzymes degraded the cell walls, and effectively controlled the growth of phytopathogenic *phytophthora* spp., the causative agent of root rot diseases in *C. obtusa* seedlings. Consequently, treatment with *B. velezensis* CE 100 suppressed the prevalence of *phytophthora* root rot diseases and enhanced the survival rate of *C. obtusa* seedlings in the greenhouse. In addition, the secretion of auxin, such as IAA by *B. velezensis* CE 100 could have increased the growth of *C. obtusa* seedlings through cell expansion and differentiation, resulting in increased growth and biomass production. Therefore, this study demonstrates that *B. velezensis* CE 100 is reliable biocontrol agent against *Phytophthora* spp., and could be effectively used in the management of root rot diseases, as well as the growth promotion of *C. obtusa* seedlings.

## Figures and Tables

**Table 1:** Average survival rates of *C. obtusa* seedlings under the different treatment groups from November 2019 to June 2020 in a greenhouse.

Treatment	Survival Rate (%)							
	2019		2020					
	November	December	January	February	March	April	May	June
Control	100 ± 0.0 ^a^	85.0 ± 5.0 ^b^	78.3 ± 5.8 ^b^	66.7 ± 7.6 ^b^	51.7 ± 7.6 ^b^	46.7 ± 3.5 ^b^	45.0 ± 8.7 ^b^	41.7 ± 5.8 ^b^
Chemical fertilizer	100 ± 0.0 ^a^	86.7 ± 2.9 ^b^	81.7 ± 5.8 ^b^	73.3 ± 2.9 ^b^	60.0 ± 5.0 ^b^	58.3 ± 7.6 ^b^	53.3 ± 5.8 ^b^	48.3 ± 5.8 ^b^
Bacterial inoculation	100 ± 0.0 ^a^	100 ± 0.0 ^a^	100 ± 0.0 ^a^	91.7 ± 2.9 ^a^	90.0 ± 5.0 ^a^	85.0 ± 5.0 ^a^	81.7 ± 3.5 ^a^	81.7 ± 3.5 ^a^

Letter indicates a significant difference between treatments significant at *p* < 0.01 by LSD test.

**Table 2:** Growth and biomass of *C. obtusa* seedlings in the control and inoculation with *B. velezensis* CE 100 treatments.

Treatment	Seedling Growth			Seedling Biomass	
	Root Collar Diameter (mm)	Shoot Length (cm)	Root Length (cm)	Shoot Dry Weight (kg)	Root Dry Weight (kg)
Control	2.4 ± 0.3 ^b^	23.8 ± 2.3 ^b^	10.3 ± 0.9 ^c^	13.8 ± 1.7 ^b^	3.1 ± 0.2 ^c^
Chemical fertilizer	2.6 ± 0.4 ^b^	25.0 ± 1.7 ^b^	12.4 ± 1.4 ^b^	14.3 ± 1.5 ^b^	3.7 ± 0.4 ^b^
Bacterial inoculation	3.2 ± 0.2 ^a^	32.6 ± 1.5 ^a^	15.5 ± 0.9 ^a^	19.6 ± 1.2 ^a^	5.8 ± 0.3 ^a^

Letter indicates a significant difference between treatments significant at *p* < 0.01 by LSD test.

## Data Availability

Data are available on request from the corresponding author.
